# The prognostic value of preoperative fibrinogen-to-prealbumin ratio and a novel FFC score in patients with resectable gastric cancer

**DOI:** 10.1186/s12885-020-06866-6

**Published:** 2020-05-06

**Authors:** Shuli Tang, Lin Lin, Jianan Cheng, Juan Zhao, Qijia Xuan, Jiayue Shao, Yang Zhou, Yanqiao Zhang

**Affiliations:** 1 Department of Medical Oncology, Harbin Medical University Cancer Hospital, No. 150, Haping Road, Nangang District, Harbin, 150001 China; 2 Department of Radiology, Harbin Medical University Cancer Hospital, No. 150, Haping Road, Nangang District, Harbin, 150001 China

**Keywords:** Gastric cancer, Inflammation, Fibrinogen-to-albumin ratio, Fibrinogen-to-prealbumin ratio, FFC score, Prognostic factor

## Abstract

**Background:**

Chronic inflammation is considered as a hallmark of gastric cancer (GC) and plays a critical role in GC progression and metastasis. This study aimed to explore the prognostic values of preoperative fibrinogen-to-prealbumin ratio (FPR), fibrinogen-to-albumin ratio (FAR), and novel FPR-FAR-CEA (FFC) score in patients with GC undergoing gastrectomy.

**Methods:**

A total of 273 patients with resectable GC were included in this retrospective study. We performed Kaplan-Meier and Cox regression analyses to assess the prognostic role of preoperative FPR, FAR, and FFC score in patients with GC and analyze their relationships with clinicopathological features.

**Results:**

Receiver operating characteristic curve (ROC) analysis revealed that the optimal cutoff values for FPR and FAR were 0.0145 and 0.0784, respectively. The FFC score had a higher area under the ROC curve than FAR and CEA. Elevated FPR (≥ 0.0145) and FAR (≥ 0.0784) were significantly associated with old age, large tumor size, tumor invasion depth, lymph nodes metastasis, advanced TNM stage, large Borrmann type, and anemia status. Kaplan-Meier analysis showed that high FPR, FAR, and FFC score were related to poor survival. Multivariate analyses indicated that FPR, FFC score, TNM stage, and tumor size were significant independent factors for survival.

**Conclusions:**

Preoperative FPR and FFC score could be used as prospective noninvasive prognostic biomarkers for resectable GC.

## Background

Gastric cancer (GC) is an important healthcare challenge worldwide, especially in eastern Asia. Recent estimates have indicated that GC is the fifth most common cancer and third leading cause of cancer-related mortality worldwide, with 1,000,000 newly diagnosed cases and 783,000 deaths annually [1]. In China, GC is the second leading cause of cancer mortality [2]. Despite new and available diagnostic and therapeutic strategies developed over the past several decades, prognosis for patients with GC remains poor with an overall 5-year relative survival rate of about 20% [3]. Although TNM classification has been clinically recognized as a strong biomarker for predicting the prognosis of GC, high heterogeneity status leads to different outcomes among patients with GC even with the same TNM stage and treatments. Thus, novel effective and noninvasive prognostic biomarkers should be identified to provide information for personalized treatment and improve patient’s outcomes.

Increasing evidence suggests that systemic inflammatory plays a critical role in cancer progression and metastasis, thereby demanding individualized immune-related therapeutic strategies [3, 4]. Recently, extensive attention has been given to systemic inflammation-based biomarkers in various malignancies. To date, several inflammation-based biomarkers, such as C-reactive protein, albumin, neutrophil-to-lymphocyte ratio (NLR), platelet-to-lymphocyte ratio (PLR), lymphocyte-to-monocyte ratio (LMR), and Glasgow prognostic score (GPS), have been revealed as useful prognostic biomarkers in GC, colorectal cancer (CRC), breast cancer, and lung cancer [5–7]. In addition, high levels of fibrinogen-to-albumin ratio (FAR), a novel inflammation-based biomarker, are associated with poor outcomes in various cancers, including esophageal squamous cell carcinoma (ESCC), breast cancer, gallbladder cancer, and soft tissue sarcoma [8, 9]. More recently, the fibrinogen-to-prealbumin ratio (FPR) was demonstrated to be an excellent diagnostic and prognostic biomarker for patients with CRC and a tool to identify individuals who can benefit from adjuvant chemotherapy treatment [10]. However, the prognostic values of FPR and FAR in GC remain unclear. Thus, in this study, we comprehensively explored the prognostic values of serum-based preoperative FPR and FAR in GC and analyzed the associations between these biomarkers and clinicopathological features. Moreover, we established a novel FPR-FAR-CEA (FFC) score based on inflammation as an independent predictor for patients with resectable GC.

## Methods

### Patient population

A total of 273 patients with resectable GC between December 2009 and December 2011 at Harbin Medical University Cancer Hospital were assessed in our study. Patients included in the study met the following criteria: (1) patients with newly histologically confirmed gastric noncardia adenocarcinoma; (2) had no distant metastatic lesions before surgery; (3) had no evidence of other malignancies; (4) had no history of autoimmune disorders, hepatitis, HIV infection, or other recent infection; (5) received curative resection; (6) had not undergone neoadjuvant chemotherapy before surgery; and (7) had complete medical records and available follow-up data. All eligible patients gave written informed consent, and our study was approved by the Institutional Ethics Committee of Harbin Medical University Cancer Hospital.

### Clinical data collection and follow-up investigation

Baseline data, including gender, age, tumor size, pathological differentiation, Borrmann type, body mass index (BMI), anemia status, and preoperative laboratory data (CEA and CA199 levels, serum fibrinogen, prealbumin, and albumin values) were gathered from medical records. All laboratory data were obtained from routine blood testing within 48 h after the first hospitalization. The pathological tumor stage (pTNM), tumor invasion depth, and lymph nodes metastasis of gastric cancers were identified according to the 7th edition AJCC TNM classification. Follow up of postoperative patients with GC was performed regularly until December 31, 2016 (more than 5 years) or until death. Patients with follow-up periods that were less than 30 days were excluded from the analysis. Overall survival (OS) was calculated from the time of operation to death or last follow up.

### Definition of inflammation-based indicators and a novel prognostic score

FPR was calculated as the serum fibrinogen value divided by the serum prealbumin value. FAR was defined as the serum fibrinogen value divided by the serum albumin value. FFC score, a novel inflammation-associated prognostic score, was established to further investigate the prognostic values of FPR, FAR, and CEA in our study. The FFC score was defined as the combination of elevated FPR, FAR, and CEA; scores of 0, 1, 2, or 3 were allocated based on the number of elevated levels. According to the optimal cutoff values for FPR and FAR were 0.0145 and 0.0784, respectively. Higher or lower levels of FPR or FAR than cutoff values were scored with 1 or 0, respectively. Patients with elevated CEA (> 5 ng/ml) or decreased CEA (≤ 5 ng/ml) were considered 1or 0 point, respectively.

### Statistical analysis

Receiver operating characteristic (ROC) curve analysis was performed to determine the optimal cut-off values of FAR and FPR based on maximum Youden index. The area under the receiver operating characteristic curve (AUC) was calculated to evaluate survival prediction. The differences between the AUC values were compared using DeLong test. Permutation test was performed to validate FFC score and avoid overfitting. Chi-square (χ^2^) test or Fisher’s exact test was performed to analyze the associations between different FPR, FAR and CEA groups and clinicopathological features. The differences among groups were estimated by Mann-Whitney U test or Kruskal-Wallis test. Survival curves were evaluated and compared with each other using the Kaplan-Meier method and log-rank test to characterize the associations between inflammation-based indicators and OS. The Cox proportional hazards model was employed for univariate and multivariate analyses to assess the independent prognostic predictor for OS. Two-sided *p* <  0.05 was considered statistically significant. All the statistical analyses were performed using SPSS version 20.0 (IBM Corp., Armonk, NY, USA) software and R project version 3.6.1.

## Results

### Baseline characteristics of patients with GC

A total of 273 patients with GC, ranging from 31 years old to 82 years old (median age of 59 years), were enrolled in the study on the basis of the inclusion criteria. All demographic and clinicopathological characteristics of patients with resectable GC are shown in Table 1 and Additional file 1: Table S1. Majority of patients were male (72.2%) and had a poorly differentiated tumor (70.0%). Over half of the patients (59.0%) had tumor size less than 5 cm, and 59.3% had lymph node metastases. A total of 89 (32.6%) and 184 (67.4%) patients were confirmed as T1 + 2 and T3 + 4 depth, respectively. According to the 8th edition of AJCC/UICC TNM classification, 66 (24.2%), 78 (28.6%), and 129 (47.2%) were diagnosed as TNM stages I, II, and III, respectively. Adjuvant chemotherapy was performed in 172 patients. A total of 86 (31.5%) patients had BMI less than 24, and 144 (52.7%) patients died during the follow-up period. The median OS was 53.87 months. On the basis of Borrmann type, the number of patients with 0-IV GC types were 46 (16.8%), 39 (14.3%), 37 (13.6%), 130 (47.6%), and 21 (7.7%), respectively. Fifty-two (19.0%) patients had elevated CEA level, 47 (17.2%) patients had elevated CA199 level, and 77 (28.2%) patients developed anemia during the study.

**Table 1:** Baseline characteristics of gastric cancer patients

Characteristic	Cases	Percentage (%)
Gender		
Male	197	72.2
Female	76	27.8
Age (years)		
< 60	152	55.7
≥ 60	121	44.3
Tumor size (cm)		
< 5	161	59.0
≥ 5	112	41.0
Pathological differentiation		
Moderate+Well	82	30.0
Poor	191	70.0
Invasion depth		
T1 + T2	89	32.6
T3 + T4	184	67.4
Lymph nodes metastasis		
Negative	111	40.7
Positive	162	59.3
TNM stage		
I	66	24.2
II	78	28.6
III	129	47.2
CEA (ng/ml)		
≤ 5	221	81.0
> 5	52	19.0
CA199 (U/ml)		
≤ 37	226	82.8
> 37	47	17.2
FPR median (range)	0.0134 (0.0038–0.1258)	
FAR median (range)	0.0760 (0.0261–0.2989)	

*CEA* carcinoembryonic antigen; *CA199 *carbohydrate antigen 199; *OS* overall survival; *FPR* fibrinogen-to-prealbumin ratio; *FAR* fibrinogen-to-albumin ratio

### Optimal cutoff values for FPR and FAR

**Fig. 1:**
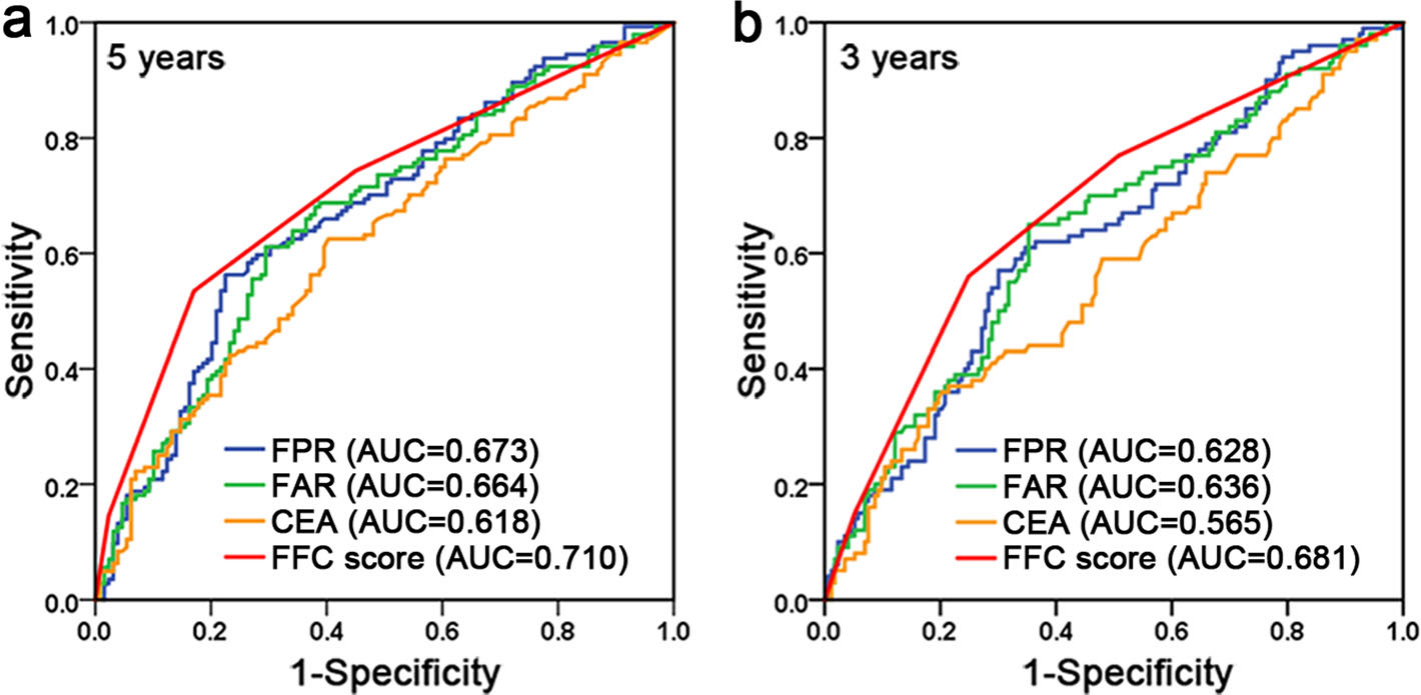
ROC curve analyses of the FPR, FAR, CEA and FFC score for 5-year (**a**) and 3-year (**b**) OS in gastric cancer. Abbreviations: ROC, receiver operating characteristic; AUC, area under ROC curve; OS, overall survival; FPR, fibrinogen-to-prealbumin ratio; FAR, fibrinogen-to-albumin ratio; CEA, carcinoembryonic antigen; FFC, FPR-FAR-CEA

As shown in Table 1, the median value of preoperative FPR was 0.0134 (range: 0.0038–0.1258), and the median value of preoperative FAR was 0.0760 (range: 0.0261–0.2989). The ROC curve and AUC for OS were computed to determine the optimal cutoff values for FPR and FAR (Fig. 1). According to the ROC curve for 5-year OS (Fig. 1a, Additional file 2: Table S2), the optimal cutoff values for FPR and FAR were 0.0145 (AUC = 0.673, *p* <  0.001, 95% confidence interval [CI]: 0.609–0.737, sensitivity = 56.3%, specificity = 77.5%), and 0.0784 (AUC = 0.664, *p* <  0.001, 95% CI: 0.600–0.728, sensitivity = 61.1%, specificity = 70.5%), respectively. The AUC values of FFC score and CEA were 0.710 (*p* <  0.001, 95% CI: 0.648–0.771) and 0.618 (*p* = 0.001, 95% CI: 0.552–0.685), respectively. Moreover, FFC score was confirmed to be no overfitting by permutation test (Additional file 3: Figure S1). The comparisons of AUC values using Delong test revealed that there were statistical differences between either FAR (*p* = 0.025) or CEA (*p* = 0.019) and FFC score. Nonetheless, there was no difference between FFC score and FPR (*p* = 0.125). In addition, as shown in Fig. 1b, the AUC values of the ROC curves for 3-year OS of FPR, FAR, CEA and FFC score were 0.628 (*p* <  0.001, 95% CI: 0.560–0.697), 0.636 (*p* <  0.001, 95% CI: 0.567–0.704), 0.565 (*p* = 0.075, 95% CI: 0.493–0.637), and 0.681 (*p* <  0.001, 95% CI: 0.615–0.748), respectively. The Delong test revealed that there were statistical differences in the AUC values between either FPR (*p* = 0.012), FAR (*p* = 0.029) or CEA (*p* = 0.003) and FFC score.

### Correlations of preoperative FPR and FAR with clinicopathological features in GC

On the basis of the cutoff values, the patients were divided into low (< 0.0145) or high (≥ 0.0145) FPR groups, low (< 0.0784) or high (≥ 0.0784) FAR groups, and low (≤ 5 ng/ml) or high (> 5 ng/ml) CEA groups. Furthermore, we evaluated the associations between these three indexes with clinicopathological features (Table 2). FPR and FAR were significantly associated with old age (*p* <  0.05), large tumor size (*p* ≤ 0.001), tumor invasion depth (*p* <  0.01), lymph node metastasis (*p* <  0.001), advanced TNM stage (*p* <  0.001), large Borrmann type (*p* <  0.01), and anemia status (*p* ≤ 0.001). Additionally, FAR was significantly related to high BMI (*p* <  0.05). However, FPR and FAR had no statistical correlations with gender, pathological differentiation, and CA199 levels (*p* > 0.05). CEA was significantly associated with tumor invasion depth (*p* <  0.001), advanced TNM stage (*p* = 0.003), and CA199 (*p* = 0.002). Nevertheless, CEA had no statistical correlations with gender, age, pathological differentiation, tumor size, lymph node metastasis, Borrmann type, BMI, and anemia status (*p* > 0.05). Mann-Whitney U test indicated that the levels of FPR and FAR in patients with T1 + 2 depth, lymph nodes metastasis-negative, and stage I were lower than in those with T3 + 4 depth, lymph nodes metastasis-positive, and stage II/III, respectively (*p* <  0.05) (Additional file 4: Figure S2).

**Table 2:** Correlation between the preoperative FPR and FAR with clinicopathological features in gastric cancer patients

Variables	FPR		***P*** value	FAR		***P*** value	CEA		***P*** value
	< 0.0145, n (%)	≥ 0.0145, n (%)		< 0.0784, n (%)	≥ 0.0784, n (%)		≤ 5 ng/ml, n (%)	> 5 ng/ml, n (%)	
Gender			0.271			1.000			1.000
Male	122 (74.8)	75 (68.2)		106 (72.1)	91 (72.2)		159 (71.9)	38 (73.1)	
Female	41 (25.2)	35 (31.8)		41 (27.9)	35 (27.8)		62 (28.1)	14 (26.9)	
Age (years)			0.013			0.002			0.277
< 60	101 (62.0)	51 (46.4)		95 (64.6)	57 (45.2)		127 (57.5)	25 (48.1)	
≥ 60	62 (38.0)	59 (53.6)		52 (35.4)	69 (54.8)		94 (42.5)	27 (51.9)	
Pathological differentiation			0.594			0.692			0.313
Moderate+Well	51 (31.3)	31 (28.2)		46 (31.3)	36 (28.6)		63 (28.5)	19 (36.5)	
Poor	112 (68.7)	79 (71.8)		101 (68.7)	90 (71.4)		158 (71.5)	33 (63.5)	
Tumor size (cm)			0.001			< 0.001			0.160
< 5	110 (67.5)	51 (46.4)		103 (70.1)	58 (46.0)		135 (61.1)	26 (50.0)	
≥ 5	53 (32.5)	59 (53.6)		44 (29.9)	68 (54.0)		86 (38.9)	26 (50.0)	
Invasion depth			0.006			< 0.001			< 0.001
T1 + T2	64 (39.3)	25 (22.8)		63 (42.9)	26 (20.6)		82 (37.1)	7 (13.5)	
T3 + T4	99 (60.7)	85 (77.2)		84 (57.1)	100 (79.4)		139 (62.9)	45 (86.5)	
Lymph nodes metastasis			< 0.001			< 0.001			0.350
Negative	81 (49.7)	30 (27.3)		75 (51.0)	36 (28.6)		93 (42.1)	18 (34.6)	
Positive	82 (50.3)	80 (72.7)		72 (49.0)	90 (71.4)		128 (57.9)	34 (65.4)	
TNM stage			< 0.001			< 0.001			0.003
I	50 (30.7)	16 (14.5)		49 (33.3)	17 (13.5)		62 (28.1)	4 (7.7)	
II	53 (32.5)	25 (22.7)		46 (31.3)	32 (25.4)		56 (25.3)	22 (42.3)	
III	60 (36.8)	69 (62.8)		52 (35.4)	77 (61.1)		103 (46.6)	26 (50.0)	
CA199 (U/ml)			0.746			1.000			0.002
≤ 37	136 (83.4)	90 (81.8)		122 (83.0)	104 (82.5)		191 (86.4)	35 (67.3)	
> 37	27 (16.6)	20 (18.2)		25 (17.0)	22 (17.5)		30 (13.6)	17 (32.7)	
Borrmann type			0.009			< 0.001			0.149
0	38 (23.3)	8 (7.3)		38 (25.9)	8 (6.3)		43 (19.5)	3 (5.8)	
I	21 (12.9)	18 (16.4)		17 (11.6)	22 (17.5)		30 (13.6)	9 (17.3)	
II	19 (11.7)	18 (16.4)		12 (8.1)	25 (19.8)		30 (13.6)	7 (13.4)	
III	71 (43.5)	59 (53.6)		68 (46.3)	62 (49.2)		100 (45.2)	30 (57.7)	
IV	14 (8.6)	7 (6.3)		12 (8.1)	9 (7.2)		18 (8.1)	3 (5.8)	
Body mass index (kg/m^2^)			0.085			0.013			0.508
< 24	105 (64.4)	82 (74.5)		91 (61.9)	96 (76.2)		149 (67.4)	38 (73.1)	
≥ 24	58 (35.6)	28 (25.5)		56 (38.1)	30 (23.8)		72 (32.6)	14 (26.9)	
Anemia status			0.001			< 0.001			0.493
Negative	130 (79.8)	66 (60.0)		119 (81.0)	77 (61.1)		161 (72.9)	35 (67.3)	
Positive	33 (20.2)	44 (40.0)		28 (19.0)	49 (38.9)		60 (27.1)	17 (32.7)	

*CEA* carcinoembryonic antigen; *CA199* carbohydrate antigen 199; *FPR* fibrinogen-to-prealbumin ratio; *FAR* fibrinogen-to-albumin ratio

### Survival analysis and prognostic impact of FPR, FAR and FFC score

**Fig. 2:**
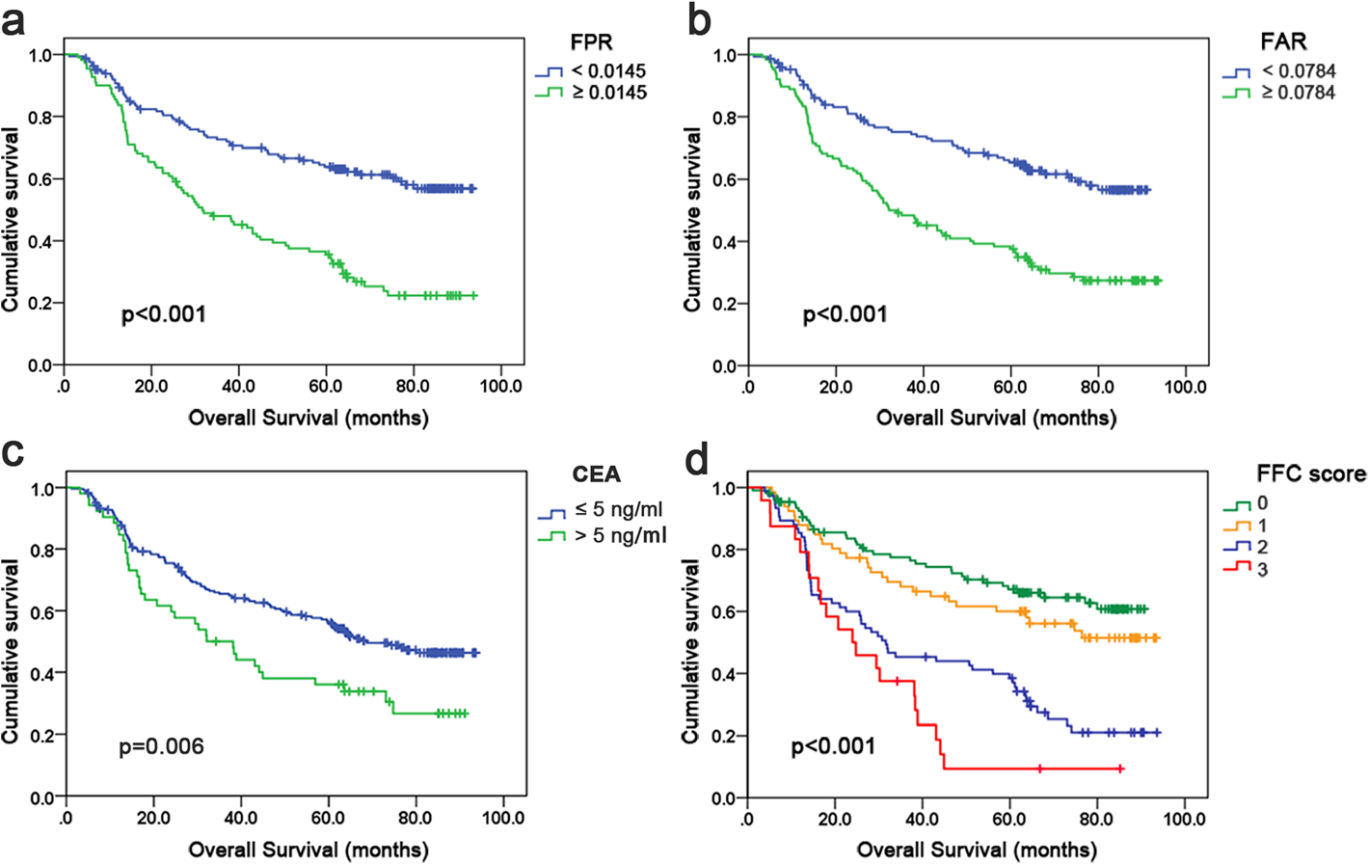
Kaplan-Meier curves for OS according to the optimal cutoff value of FPR (**a**), FAR (**b**), CEA (**c**) and FFC score (**d**) in gastric cancer patients. Abbreviations: OS, overall survival; FPR, fibrinogen-to-prealbumin ratio; FAR, fibrinogen-to-albumin ratio; CEA, carcinoembryonic antigen; FFC, FPR-FAR-CEA

In this study, Kaplan-Meier analysis was conducted to identify the prognostic significance of FPR, FAR, and FFC score. As revealed in Fig. 2, short OS was demonstrated to be significantly associated with high levels of FPR (≥ 0.0145; Fig. 2a; *p* <  0.001), FAR (≥ 0.0784; Fig. 2b; *p* <  0.001), CEA (> 5 ng/ml; Fig. 2c; *p* = 0.006), and FFC score (≥ 1; Fig. 2d; *p* <  0.001). Additionally, patients with FFC score = 3 experienced the lowest survival rate compared with those belonging to the three other groups. Conversely, patients with FFC score = 0 had the highest OS among the four groups. Kruskal-Wallis test indicated that there was no significant difference in terms of prediction of survival in the three different situations of FFC score = 2 (*p* = 0.812) (Additional file 5: Figure S3).

Cox proportional hazards model was selected for further survival analysis to evaluate the prediction models of baseline characteristics and preoperational FPR, FAR, and FFC score. As shown in Table 3, FPR (HR = 2.499, 95% CI: 1.794–3.483, *p* <  0.001), FAR (HR = 2.343, 95% CI: 1.673–3.280, *p* <  0.001), lymph node metastasis (HR = 5.119, 95% CI: 3.296–7.952, *p* <  0.001), TNM stage (HR = 3.147, 95% CI: 2.385–4.151, *p* <  0.001), Borrmann type (HR = 1.433, 95% CI: 1.238–1.658, *p* <  0.001), tumor size (HR = 3.587, 95% CI: 2.559–5.028, *p* <  0.001), anemia status (HR = 1.512, 95% CI: 1.071–2.134, *p* = 0.019), CEA (HR = 1.686, 95% CI: 1.155–2.462, *p* = 0.007), and CA199 (HR = 1.648, 95% CI: 1.106–2.455, *p* = 0.014) were determined as statistically significant prognostic factors for OS by univariate analysis. However, OS had no associations with age, gender, and pathological differentiation.

**Table 3:** Univariate and multivariate analysis for OS in gastric cancer patients

Variables	Univariate analysis		Multivariate analysis	
	HR (95%CI)	***P*** value	HR (95%CI)	***P*** value
Gender	0.742 (0.505–1.091)	0.129		
Age	1.270 (0.916–1.761)	0.152		
Pathological differention	1.191 (0.909–1.562)	0.205		
Lymph nodes metastasis	5.119 (3.296–7.952)	< 0.001	1.646 (0.864–3.135)	0.130
TNM stage	3.147 (2.385–4.151)	< 0.001	3.564 (1.321–9.618)	0.012
Borrmann type	1.433 (1.238–1.658)	< 0.001	0.980 (0.808–1.187)	0.834
Tumor size	3.587 (2.559–5.028)	< 0.001	1.958 (1.349–2.842)	< 0.001
Body mass index	0.884 (0.619–1.264)	0.500		
Anemia status	1.512 (1.071–2.134)	0.019	0.971 (0.676–1.394)	0.872
CEA	1.686 (1.155–2.462)	0.007	1.422 (0.959–2.107)	0.080
CA199	1.648 (1.106–2.455)	0.014	1.043 (0.689–1.579)	0.842
FPR	2.499 (1.794–3.483)	< 0.001	1.595 (1.072–2.373)	0.021
FAR	2.343 (1.673–3.280)	< 0.001	1.236 (0.829–1.842)	0.299
FFC score	1.704 (1.446–2.009)	< 0.001	1.414 (1.189–1.683)	< 0.001

*OS* overall survival; *HR* hazard ratio; *CI* confidence interval; *CEA* carcinoembryonic antigen; *CA199* carbohydrate antigen 199; *FPR* fibrinogen-to-prealbumin ratio; *FAR* fibrinogen-to-albumin ratio; *FFC* FPR-FAR-CEA

To further assess the independent prognostic predictor for OS, the factors with a *p* value < 0.1 in the univariate analysis were enrolled in the multivariate Cox proportional hazards model. Multivariate analysis showed FPR (HR = 1.595, 95% CI: 1.072–2.373, *p* = 0.021), TNM stage (HR = 3.564, 95% CI: 1.321–9.618, *p* = 0.012), and tumor size (HR = 1.958, 95% CI: 1.349–2.842, *p* <  0.001) were significant independent predictors of OS. However, FAR, CEA, CA199, lymph node metastasis, Borrmann type, and anemia status had no significance in multivariate analysis.

The Cox proportional hazards model based on lymph node metastasis, TNM stage, Borrmann type, tumor size, anemia status and CA199 was used to further confirm the prognostic value of the FFC score. As shown in Table 3, univariate analysis showed that the FFC score is a significant prognostic factor (HR = 1.704, 95% CI: 1.446–2.009, *p* <  0.001). The FFC score was also a significant independent predictor in multivariate analysis (HR = 1.414, 95% CI: 1.189–1.683, *p* <  0.001).

### Subgroup analysis

**Fig. 3:**
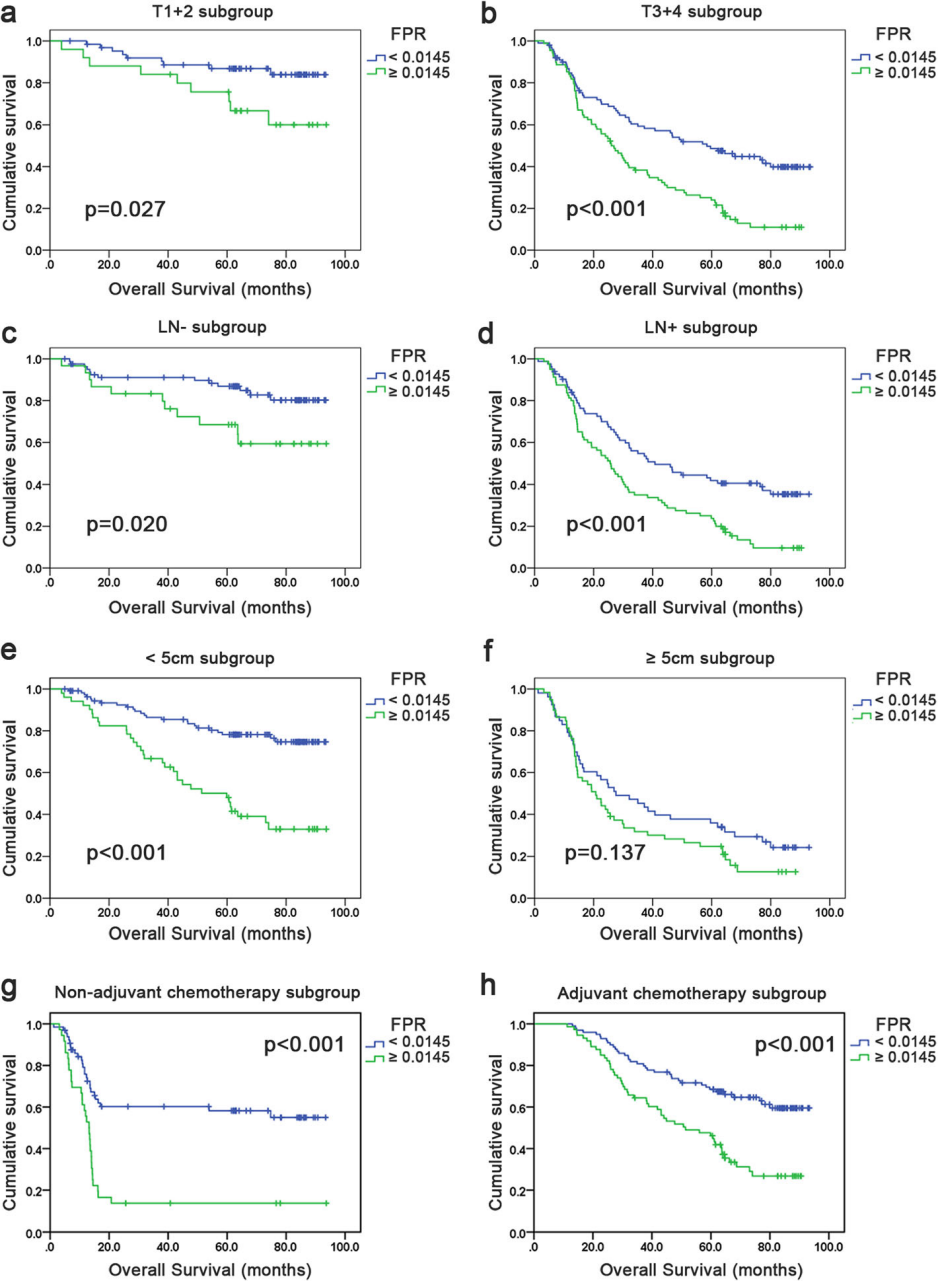
Kaplan-Meier curves analyses for OS according to the optimal cutoff value of FPR in each subgroup. **a** T1 + 2 subgroup; **b** T3 + 4 subgroup; **c** LN- subgroup; **d** LN+ subgroup; **e** tumor size < 5 cm subgroup; **f** tumor size ≥5 cm subgroup; **g** non-adjuvant chemotherapy subgroup; **h** adjuvant chemotherapy subgroup. Abbreviations: OS, overall survival; FPR, fibrinogen-to-prealbumin ratio; LN-, lymph nodes metastasis-negative; LN+, lymph nodes metastasis-positive

Individual subgroup analyses were performed based on tumor invasion depth, lymph node metastasis, adjuvant chemotherapy, and tumor size to investigate the prognostic significance of FPR, and FFC score in patients with GC. As shown in Fig. 3, extended OS was found in patients with low FPR in the T1 + 2 and T3 + 4 subgroups (*p* = 0.027 and *p* <  0.001, respectively), lymph nodes metastasis-negative and -positive subgroups (*p* = 0.020 and *p* <  0.001, respectively), non-adjuvant chemotherapy and adjuvant chemotherapy subgroups (*p* <  0.001), and <  5 cm subgroup (*p* <  0.001), but not in the ≥5 cm subgroup (*p* = 0.137). In addition, short OS was found in patients with high FFC score (≥ 2) in the T1 + 2 and T3 + 4 subgroups (*p* = 0.021 and *p* <  0.001, respectively), lymph nodes metastasis-negative and -positive subgroups (*p* <  0.001), non-adjuvant chemotherapy and adjuvant chemotherapy subgroups (*p* <  0.001), and <  5 cm subgroup (*p* <  0.001), but not in the ≥5 cm subgroup (*p* = 0.093) (Additional file 6: Figure S4). The subgroup analyses based on TNM for FPR, FAR and FFC score were also performed in our study. Short OS was found in high FPR, FAR and FFC score (≥ 2) in both stage I and III subgroup (*p* <  0.05). While, there were no statistically significant difference in OS in high or low FPR, FAR and FFC score in the stage II subgroup (*p* > 0.05) (Additional file 7: Figure S5). In this study, we also evaluated FFC score in high FPR group (≥ 0.0145), and we found the OS in patients with high FFC score was shorter (*p* = 0.019) (Additional file 8: Figure S6).

## Discussion

The association between GC and chronic inflammation has attracted extensive attention over the past decades. Currently, chronic inflammation has been recognized as a hallmark of GC and can occur in GC initiation and progression [11]. For instance, *Helicobacter pylori* infection triggers gastric carcinogenesis with a unique and complex process involving chronic atrophic gastritis, intestinal metaplasia, and occurrence of invasive carcinoma [12, 13]. Emerging evidence indicated that immune cells and inflammatory cytokines play vital roles in the progression of GC by regulating the tumor microenvironment [14, 15]. Furthermore, understanding inflammatory mechanisms in GC will establish personalized immune-related treatment against cancer and identify novel diagnostic and prognostic biomarkers for patients with GC [16]. Consequently, chronic inflammation plays a key role in GC, and inflammation-associated factors provide a rich resource for biomarkers.

In recent years, numerous studies described the essential role of inflammation-related markers in the diagnosis and prognosis for GC. For example, elevated NLR and PLR and decreased LMR in patients with both early and advanced GC are related to poor prognosis [4, 5, 17, 18]. Similarly, patients with GC and high AGR present poor survival compared with those with low AGR [19]. Many inflammation-related markers have been explored as a prognostic predictor for GC; however, additional reliable indices are still required.

Fibrinogen is a pivotal coagulation-related protein in mediating communication between hemostatic components and cancer biology. Tumor progression and metastasis can be promoted by fibrinogen through different mechanisms, such as stimulation of angiogenesis, promotion of platelet adhesion, and enhancement of tumor cell proliferation and migration by binding to growth factors [20]. In addition, patients with GC and hyperfibrinogenemia have increased risk of poor clinical outcome and lymphatic metastasis, and treatment for such patients can be optimized by evaluating peripheral fibrinogen. For instance, in a large cohort of 1196 patients with GC, elevated fibrinogen was found to be positively correlated with low survival [21]. Similarly, in 1090 patients with GC who underwent surgery, preoperative fibrinogen was proposed as an independent prognostic biomarker [22]. Furthermore, circulating albumin and prealbumin are indicators for assessing nutritional status and markers of immune status. Tumor progression can be restrained by albumin by stabilizing DNA replication and enhancing immunity response [23]. Albumin is widely used as an ideal drug delivery platform in anti-inflammatory and anticancer therapy because it accumulates at inflammation and tumor sites [24, 25]. Additionally, hypoalbuminemia has been associated with poor prognosis in GC due to malnutrition and postoperative complications [26].

Fibrinogen, albumin, and prealbumin are important components of inflammation-associated GC. However, the prognostic value of their combination in GC remains unclear. In this study, we demonstrated that both preoperative FPR and FAR were promising noninvasive prognostic biomarkers for GC, and FPR was an independent prognostic predictor for resectable GC. These results were consistent with previous research on CRC [10, 27]. Increasing studies have used FAR as an independent prognostic factor in patients with resectable ESCC, hepatocellular carcinoma, and advanced non-small cell lung cancer, and high levels of FAR can lead to high risk of recurrence and unfavorable OS [8, 28, 29]. Unfortunately, FAR was not an independent prognostic predictor for resectable GC in our study. Our results suggested that FPR is a more suitable prognostic biomarker for GC than FAR.

In subgroup analysis, we found that short OS was related to high FPR and FFC score in patients with GC across all depths, lymph nodes status, and AJCC stage subgroups. For patients with a tumor size of ≥5 cm, FPR and FFC score were not related to OS. These findings suggested that both FPR and FFC score were highly useful predictors for small tumors in GC.

Similar to a single inflammatory biomarker, emerging prognostic scores based on immune and nutritional status are attracting attention as novel and superior predictors of prognosis in GC. For instance, GPS or modified GPS based on cancer-related inflammation is an independent predictor of GC survival and can be used as a predictor for patients with GC undergoing platinum-based chemotherapy [30, 31]. Moreover, low prognostic nutritional index (PNI), which is calculated from the combination of BMI, lymphocyte count, and albumin, is associated with poor clinical outcome in resectable GC and can act as an independent risk factor [32]. Similarly, controlling nutritional status (CONUT) score, which is obtained from total lymphocyte count, total cholesterol level, and albumin, is associated with survival in patients with GC undergoing curative gastrectomy [33]. Despite extensive work on prognostic scores, additional effective and reliable prognostic scores need to be investigated. In this study, we demonstrated that FFC score was superior to FAR or CEA, and considered an independent prognostic factor for GC. In addition, short OS was found in patients with high FFC score in high FPR group (*p* = 0.019). It indicated that FFC score could be used for further stratification for high FPR group. Compared to purely using FPR, FFC score could provide more precise prognostic information for the patients with high FPR.

In recent years, with the substantial progress and clinical application of artificial intelligence, machine learning along with the explosive growth of biomedical big-data has generated much interest in developing clinical informatics tools for disease diagnosis, staging, and prognosis [34]. There was a strong power in machine learning approach on constructing new scoring system based on existing clinical indicators. Despite some scoring systems for cancer have been created and validated by the novel method of machine learning [35], the evidence of scoring system based on inflammation biomarkers for gastric cancer is still lacking and elusive. Thus, more studies using the novel method of machine learning are needed to discover more effective biomarker in gastric cancer.

This study was the first to establish FFC score, a novel scoring system based on FPR, FAR, and CEA, and investigate its prognostic value in resectable GC. However, our study still had several potential limitations. First, cases enrolled in the retrospective study were from a single center, so sample size was limited and had selection bias. Second, diverse adjuvant chemotherapy may lead to heterogeneous clinical outcomes. Third, the consistency of peripheral blood results with tumor tissue still need further investigation. Fourth, although the optimal cutoff values was determined by ROC curves, the sensitivity of the optimal cutoff values for FPR and FAR is limited. This may due to the highly heterogeneous of gastric cancer, and a relatively small size of enrolled patients. Therefore, further research with a large cohort from multiple centers are needed to confirm the cutoff values for FPR and FAR, and validate the prognostic roles of FPR level and FFC score in GC.

## Conclusions

Preoperative FPR and FFC score, which are convenient, quick, cost-effective, and entail minimal pain, could be used as prospective noninvasive prognostic biomarkers for resectable GC. Low FPR and FFC score may improve the survival of patients with resectable GC.

## Supplementary information

**Additional file 1 Table S1.** Baseline characteristics of gastric cancer patients.

**Additional file 2 Table S2.** ROC curve analyses and optimal cutoff values of FPR and FAR for 5-year OS in gastric cancer.

**Additional file 3 Figure S1.** ROC curve analyses and permutation test of FFC score. (a) The AUC of FFC score reached 0.710 by using the median value of 1.5 as the cutoff value. (b) A 200 times permutation test for (a) showed that FFC score could be significantly distinguished from random effect.

**Additional file 4 Figure S2.** The correlation of preoperative FPR and FAR with pathological features. (a) FPR with invation depth; (b) FPR with lymph node statu; (c) FPR with pathological tumor stage; (d) FAR with invation depth; (e) FAR with lymph node statu; (f) FAR with pathological tumor stage. The comparisons between two groups were assessed using Mann-Whitney U test.

**Additional file 5 Figure S3.** Differences in overall survival in the different situations of FFC score = 2 (Kruskal-Wallis test, *p* = 0.812).

**Additional file 6 Figure S4.** Kaplan-Meier curves analyses for OS according to FFC score in each subgroup. (a) T1 + 2 subgroup; (b) T3 + 4 subgroup; (c) LN- subgroup; (d) LN+ subgroup; (e) tumor size < 5 cm subgroup; (f) tumor size ≥5 cm subgroup; (g) non-adjuvant chemotherapy subgroup; (h) adjuvant chemotherapy subgroup.

**Additional file 7 Figure S5.** Kaplan-Meier curves analyses for OS according to the optimal cutoff value of FPR, FAR, and FFC score in TNM subgroup. (a) FPR-stage I subgroup; (b) FPR-stage II subgroup; (c) FPR-stage III subgroup; (d) FAR-stage I subgroup; (e) FAR-stage II subgroup; (f) FAR-stage III subgroup; (g) FFC score-stage I subgroup; (h) FFC score-stage II subgroup; (i) FFC score-stage III subgroup.

**Additional file 8 Figure S6.** Kaplan-Meier curves analyses for OS according to FFC score in high FPR group (≥ 0.0145)

## Data Availability

The data are available from the corresponding author on reasonable request.

## Abbreviations

AUC: Area under the receiver operating characteristic curve
BMI: Body mass index
CEA: Carcinoembryonic antigen
CA199: Carbohydrate antigen 199
CI: Confidence interval
CONUT: Controlling nutritional status
CRC: Colorectal cancer
ESCC: Esophageal squamous cell carcinoma
FAR: Fibrinogen-to-albumin ratio
FPR: Fibrinogen-to-prealbumin ratio
FFC: FPR-FAR-CEA
GC: gastric cancer
GPS: Glasgow prognostic score
LMR: Lymphocyte-to-monocyte ratio
NLR: Neutrophil-to-lymphocyte ratio
OS: Overall survival
PLR: Platelet-to-lymphocyte ratio
PNI: Prognostic nutritional index
ROC: Receiver operating characteristic.
