# Supplementary material for: The prognostic value of preoperative fibrinogen-to-prealbumin ratio and a novel FFC score in patients with resectable gastric cancer
Source: BMC Cancer. 2020 May 6;20:382. doi: 10.1186/s12885-020-06866-6 (PMC7201974; doi:10.1186/s12885-020-06866-6)
Supplement: Supplementary file 1 — Additional file 1 Table S1. Baseline characteristics of gastric cancer patients. [file 12885_2020_6866_MOESM1_ESM.docx]

**Additional file 1:** **Table S1.** Baseline characteristics of gastric cancer patients.

| **Characteristic** | **Cases** | **Percentage (%)** |
| --- | --- | --- |
| Body mass index (kg/m^2^) |  |  |
| < 24 | 187 | 68.5 |
| ≥ 24 | 86 | 31.5 |
| Borrmann type |  |  |
| 0 | 46 | 16.8 |
| Ⅰ | 39 | 14.3 |
| Ⅱ | 37 | 13.6 |
| Ⅲ | 130 | 47.6 |
| Ⅳ | 21 | 7.7 |
| Anemia status |  |  |
| Negative | 196 | 71.8 |
| Positive | 77 | 28.2 |
| Adjuvant Chemotherapy |  |  |
| No | 101 | 37.0 |
| Yes | 172 | 63.0 |
| OS End-point |  |  |
| Alive | 129 | 47.3 |
| Dead | 144 | 52.7 |
| OS (months) median (range) | 53.87 (1.2-93.63) | |

OS, overall survival.
