# Supplementary material for: The prognostic value of preoperative fibrinogen-to-prealbumin ratio and a novel FFC score in patients with resectable gastric cancer
Source: BMC Cancer. 2020 May 6;20:382. doi: 10.1186/s12885-020-06866-6 (PMC7201974; doi:10.1186/s12885-020-06866-6)
Supplement: Supplementary file 2 — Additional file 2 Table S2. ROC curve analyses and optimal cutoff values of FPR and FAR for 5-year OS in gastric cancer. [file 12885_2020_6866_MOESM2_ESM.docx]

**Additional file 2:** **Table S2.** ROC curve analyses and optimal cutoff values of FPR and FAR for 5-year OS in gastric cancer.

| **Index** | **AUC** | ***P* value** | **95%CI** | **Cutoff** | **Sensitivity** | **Specificity** |
| --- | --- | --- | --- | --- | --- | --- |
| FPR | 0.673 | < 0.001 | 0.609-0.737 | 0.0145 | 0.563 | 0.775 |
| FAR | 0.664 | < 0.001 | 0.600-0.728 | 0.0784 | 0.611 | 0.705 |

ROC, receiver operating characteristic; OS, overall survival; FPR, fibrinogen-to-prealbumin ratio; FAR, fibrinogen-to-albumin ratio; AUC, area under the receiver operating characteristic curve; CI, confidence interval.
