# Supplementary figures and images for: The prognostic value of preoperative fibrinogen-to-prealbumin ratio and a novel FFC score in patients with resectable gastric cancer
Source: BMC Cancer. 2020 May 6;20:382. doi: 10.1186/s12885-020-06866-6 (PMC7201974; doi:10.1186/s12885-020-06866-6)

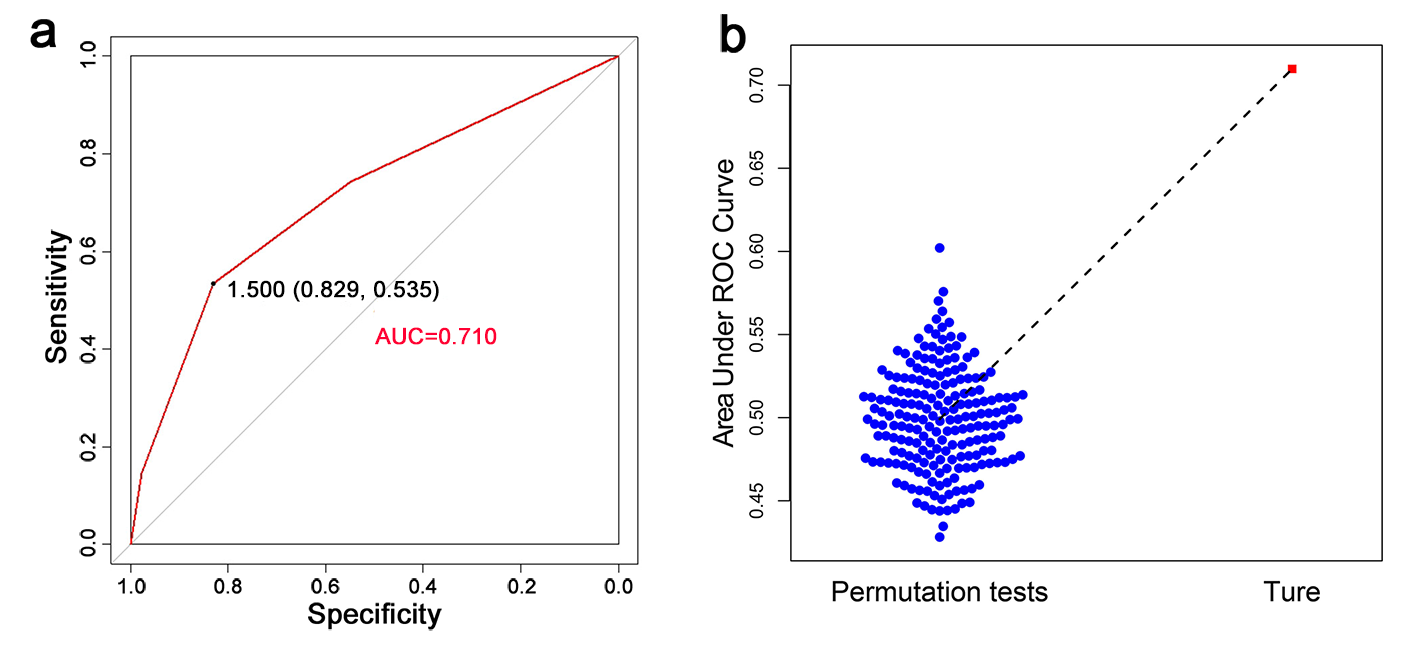

Supplement: Supplementary file 3 — Additional file 3 Figure S1. ROC curve analyses and permutation test of FFC score. (a) The AUC of FFC score reached 0.710 by using the median value of 1.5 as the cutoff value. (b) A 200 times permutation test for (a) showed that FFC score could be significantly distinguished from random effect. [file 12885_2020_6866_MOESM3_ESM.tif]

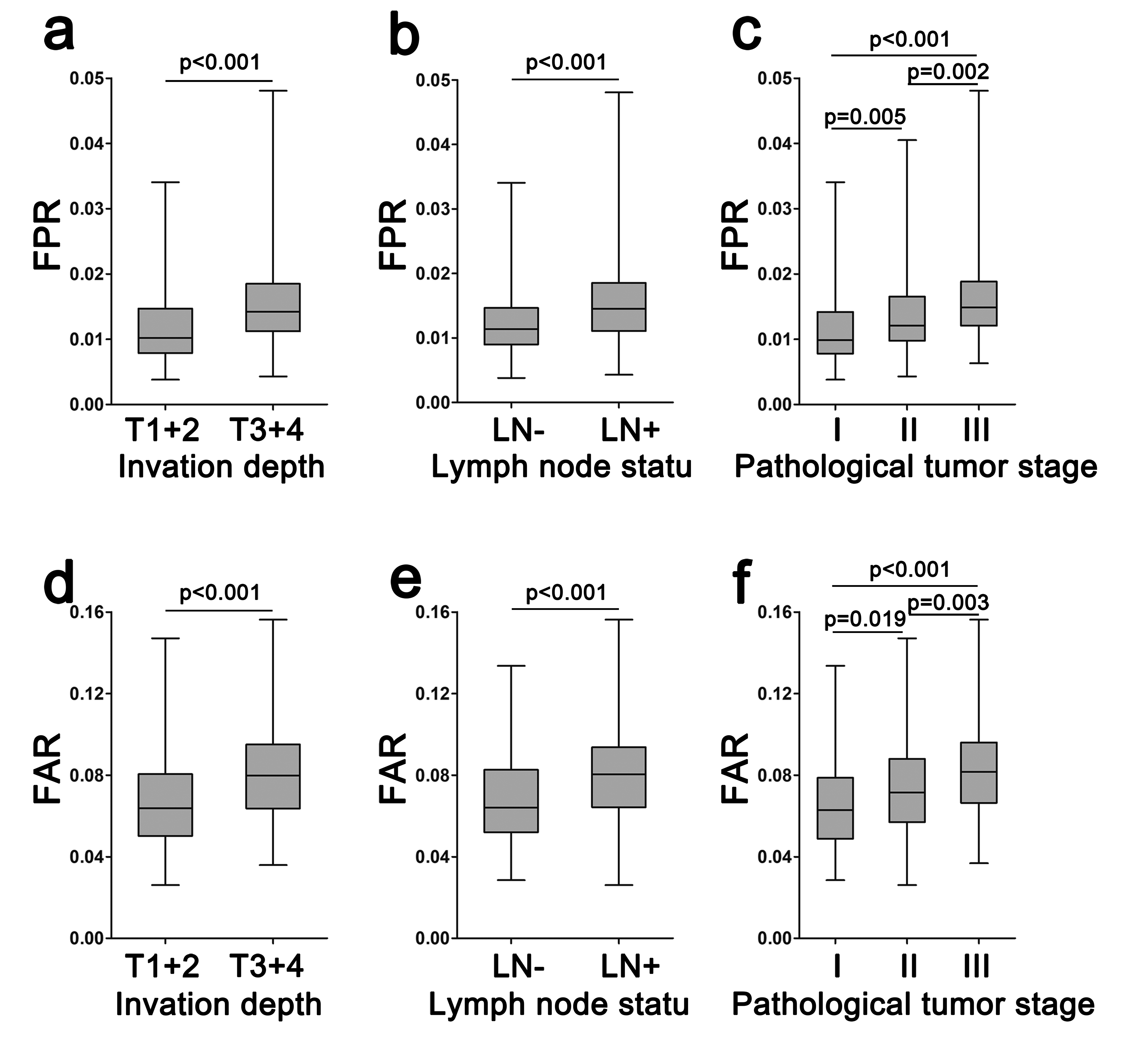

Supplement: Supplementary file 4 — Additional file 4 Figure S2. The correlation of preoperative FPR and FAR with pathological features. (a) FPR with invation depth; (b) FPR with lymph node statu; (c) FPR with pathological tumor stage; (d) FAR with invation depth; (e) FAR with lymph node statu; (f) FAR with pathological tumor stage. The comparisons between two groups were assessed using Mann-Whitney U test. [file 12885_2020_6866_MOESM4_ESM.tif]

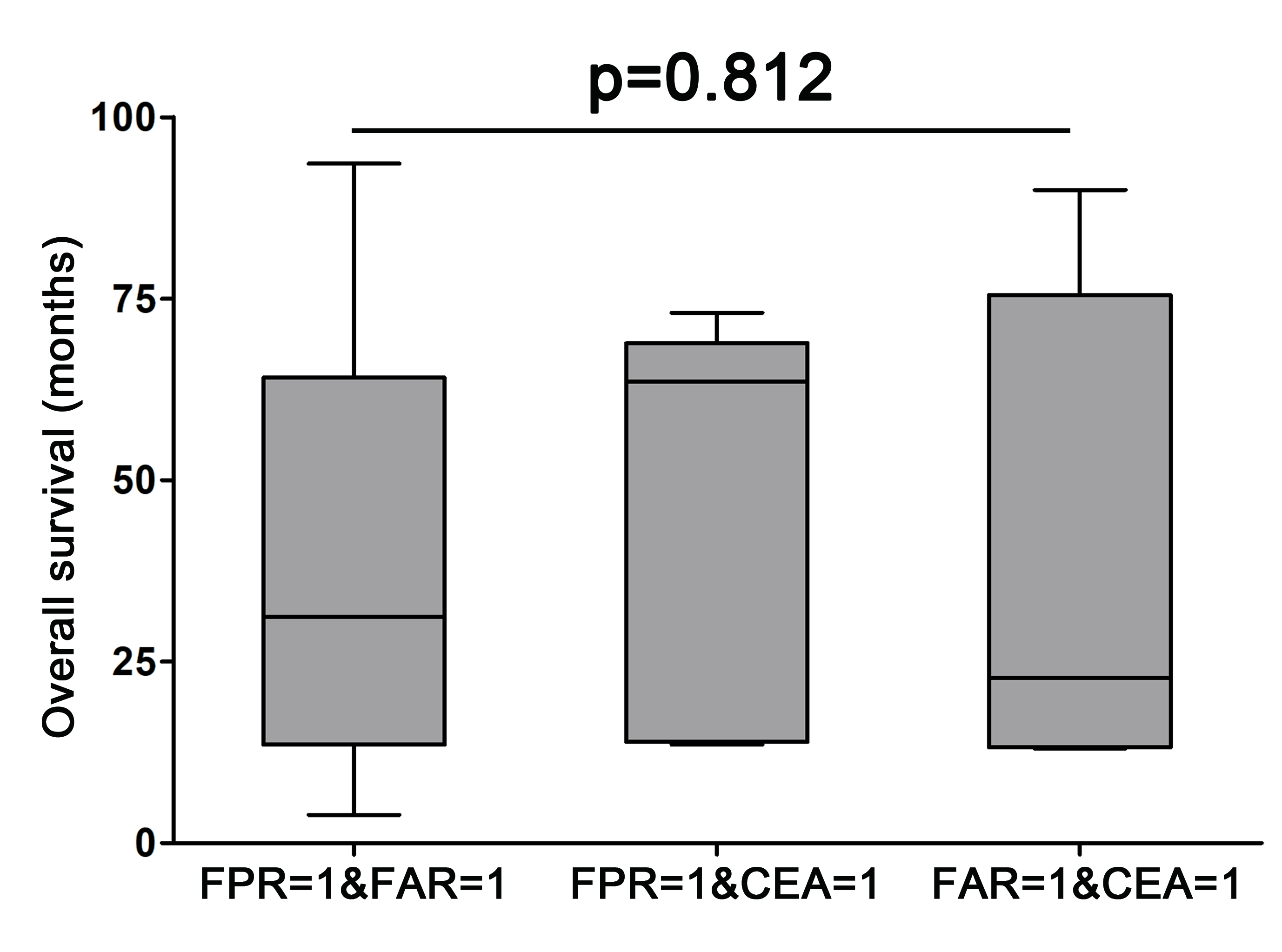

Supplement: Supplementary file 5 — Additional file 5 Figure S3. Differences in overall survival in the different situations of FFC score = 2 (Kruskal-Wallis test, p = 0.812). [file 12885_2020_6866_MOESM5_ESM.tif]

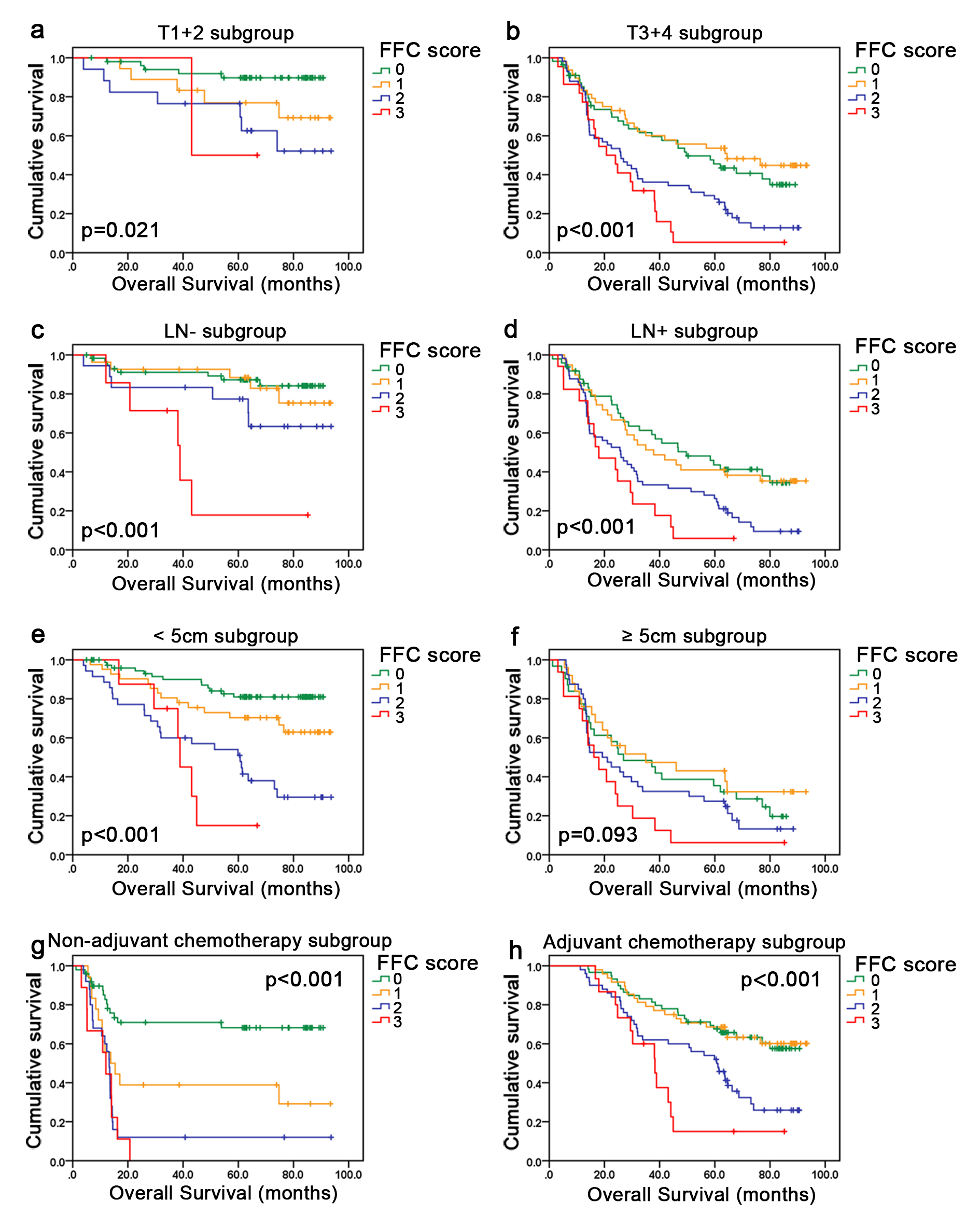

Supplement: Supplementary file 6 — Additional file 6 Figure S4. Kaplan-Meier curves analyses for OS according to FFC score in each subgroup. (a) T1 + 2 subgroup; (b) T3 + 4 subgroup; (c) LN- subgroup; (d) LN+ subgroup; (e) tumor size < 5 cm subgroup; (f) tumor size ≥5 cm subgroup; (g) non-adjuvant chemotherapy subgroup; (h) adjuvant chemotherapy subgroup. [file 12885_2020_6866_MOESM6_ESM.tif]

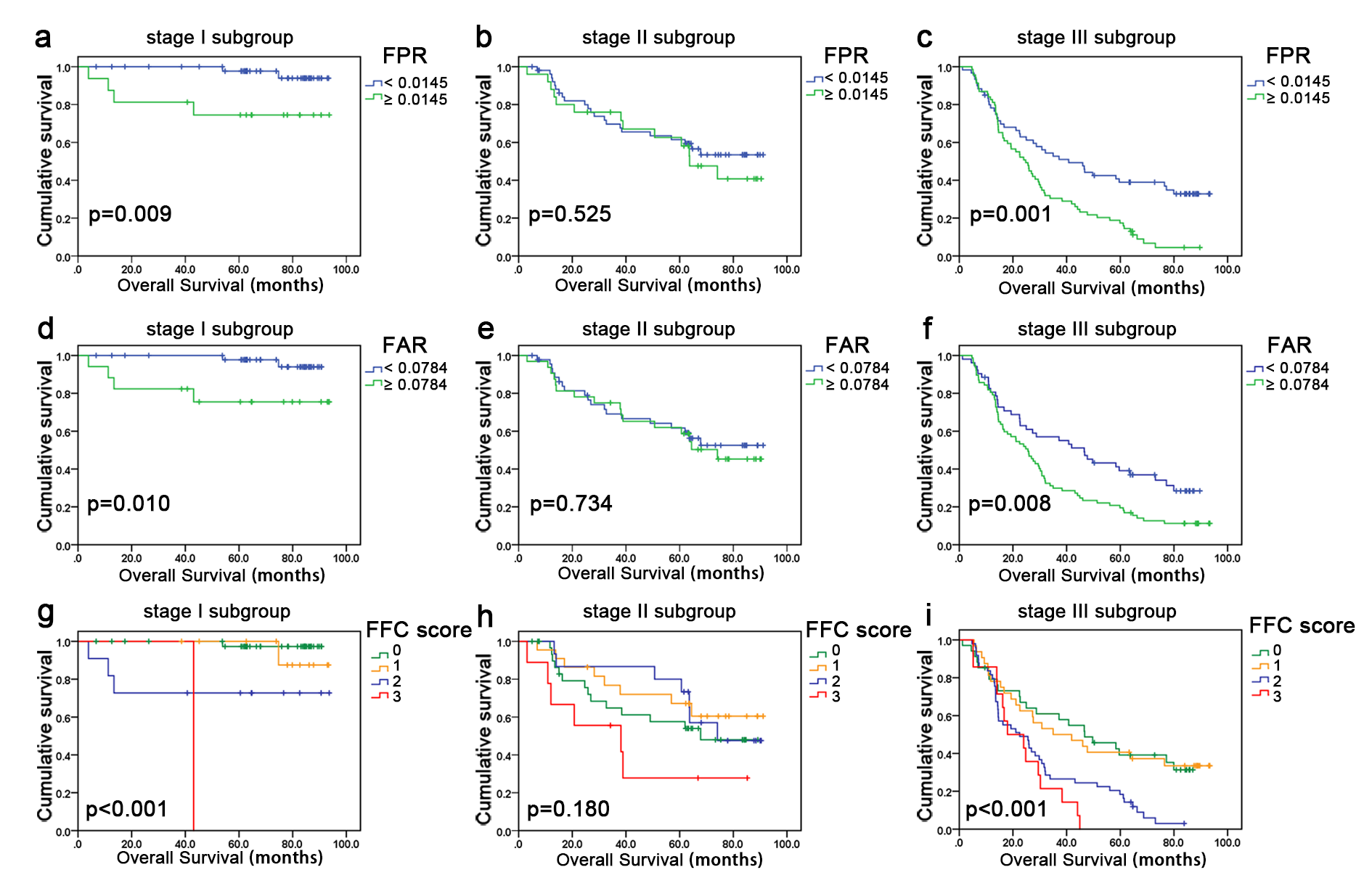

Supplement: Supplementary file 7 — Additional file 7 Figure S5. Kaplan-Meier curves analyses for OS according to the optimal cutoff value of FPR, FAR, and FFC score in TNM subgroup. (a) FPR-stage I subgroup; (b) FPR-stage II subgroup; (c) FPR-stage III subgroup; (d) FAR-stage I subgroup; (e) FAR-stage II subgroup; (f) FAR-stage III subgroup; (g) FFC score-stage I subgroup; (h) FFC score-stage II subgroup; (i) FFC score-stage III subgroup. [file 12885_2020_6866_MOESM7_ESM.tif]

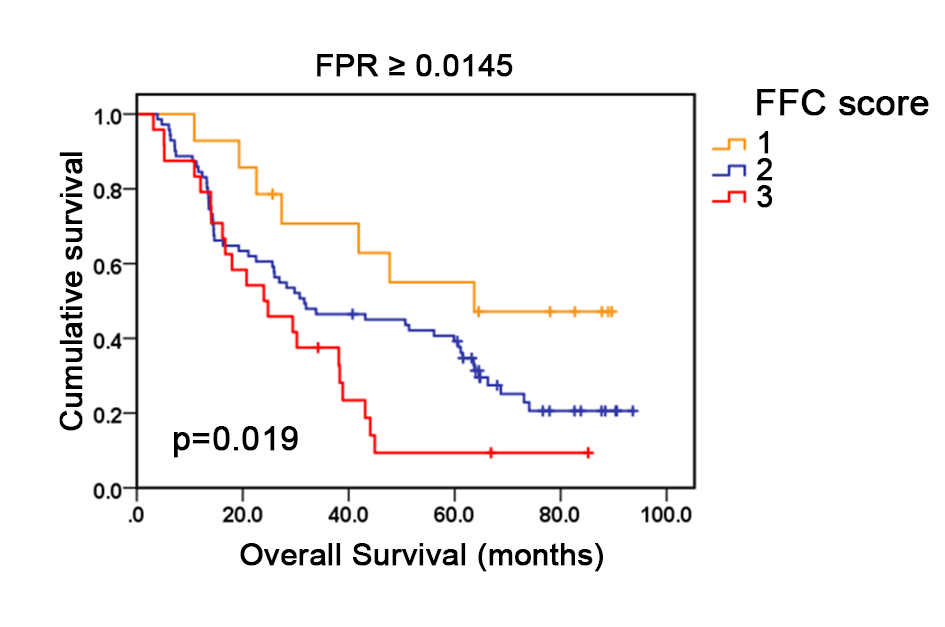

Supplement: Supplementary file 8 — Additional file 8 Figure S6. Kaplan-Meier curves analyses for OS according to FFC score in high FPR group (≥ 0.0145) [file 12885_2020_6866_MOESM8_ESM.tif]
